# Fickle Judgments in Moral Dilemmas: Time Pressure and Utilitarian Judgments in an Interdependent Culture

**DOI:** 10.3389/fpsyg.2022.795732

**Published:** 2022-03-03

**Authors:** Hirofumi Hashimoto, Kaede Maeda, Kaede Matsumura

**Affiliations:** ^1^Graduate School of Literature and Human Sciences, Osaka City University, Osaka, Japan; ^2^Urban-Culture Research Center, Osaka City University, Osaka, Japan; ^3^Japan Society for the Promotion of Science, Tokyo, Japan; ^4^Graduate School of Letters, Yasuda Women’s University, Hiroshima, Japan

**Keywords:** moral dilemma, utilitarian judgment, deontological judgment, responsibility, interdependence

## Abstract

In the trolley problem, a well-known moral dilemma, the intuitive process is believed to increase deontological judgments, while deliberative reasoning is thought to promote utilitarian decisions. Therefore, based on the dual-process model, there seems to be an attempt to save several lives at the expense of a few others in a deliberative manner. This study examines the validity of this argument. To this end, we manipulate decision-making time in the standard trolley dilemma to compare differences among 119 Japanese female undergraduates under three conditions: intuitive judgment, deliberative judgment, and judgment after a group discussion. The current results demonstrate that utilitarian judgments decreased from 52.9% in the intuition condition to 43.7% in the deliberation condition and 37.0% after the discussion. Additional analysis suggests that the decrease in utilitarian judgments may be related to psychological unwillingness to assume responsibility for the lives of others rather than to an increase in deontological judgments. Finally, these results are discussed from an adaptationist perspective.

## Introduction

In ethics, deontology and utilitarianism are understood as principles for the rightness of moral decision-making. Utilitarianism is a principle that emphasizes the consequences that actions have on people and posits that actions that lead to the greatest happiness for the greatest number are ethically right. Jeremy Bentham and John Stuart Mill are among the most prominent advocates. Utilitarianism is sometimes referred to as a species of consequentialism. In contrast, deontology, espoused by Immanuel Kant, focuses on duties defined by right and wrong. It posits that the ethical rightness of an action depends not on what consequences it brings but on the rightness of the act itself, that is, whether it is done in accordance with duty. In the “trolley problem” (Foot, 1978; Thomson, 1985), a well-known moral dilemma, people are forced to make a moral decision between these two ethical judgments, that is, harming one person (utilitarian judgments) or letting many people die (deontological judgments). Specifically, in the standard trolley dilemma, five workers working on the tracks are expected to be hit and killed by a runaway train with failed brakes. However, by pulling the lever to divert the runaway trolley onto the sidetrack, one can save the lives of the five workers in exchange for the life of another worker. Previous studies have demonstrated that, in response to this trolley dilemma, people generally deem that it is morally appropriate to pull the lever to save five lives (e.g., Greene et al., 2001). Regarding the so-called standard footbridge dilemma, such utilitarian judgments that it is morally justified to push someone off a footbridge and into the path of an out-of-control trolley are less likely to be exhibited, but rather moral reasoning shifts to deontological judgments. Past research has attempted to explain why people react differently to these two moral dilemmas—trolley and footbridge dilemmas—from multiple perspectives.

One model that explains people’s utilitarian and deontological judgments when faced with moral dilemmas is the dual-process model of thinking (Evans, 2008; Kahneman, 2011; Evans and Stanovich, 2013). According to the general explanation based on this dual-process model (e.g., Greene et al., 2001, 2008; Haidt, 2007), deontological judgments are assumed to be underpinned by System 1 thinking (the fast, automatic, and emotional process). On the other hand, utilitarian judgments are based on System 2 thinking (a slow, cognitive, and effortful process). Furthermore, the dual-process model assumes that intuition precedes deliberation; therefore, deontological judgments are explained as predating utilitarian judgments (Greene et al., 2004). This explanation is seemingly consistent with some empirical findings (see Capraro, 2019 for a review). More specifically, empirical support has been provided by a large number of research findings using functional magnetic resonance imaging (Greene et al., 2001, 2004), manipulating decision-making time (Suter and Hertwig, 2011) or cognitive load (Greene et al., 2008), and focusing on working memory (Moore et al., 2008). However, this explanation is still being debated from various perspectives and has not been sufficiently concluded. More recently, some research papers showed the conflicting findings (Tinghög et al., 2016; Baron and Gürçay, 2017; Gürçay and Baron, 2017) and others cast doubt on the assumption of the model that deontological judgment precedes utilitarian judgment (e.g., Bago and De Neys, 2019). However, these aggregated insights into “fickle” judgments in moral dilemmas have not sufficiently examined the socio-ecological environment. Therefore, the current paper examines moral judgments by focusing on two potential influencing factors: decision-making time and the socio-ecological environment.

We focus on both the dual-process theory and the socio-ecological environment because most prior studies that have applied the dual-process model to moral dilemma issues have been conducted in Western countries. However, the number of studies discussing cross-cultural differences has increased (Gold et al., 2014; Awad et al., 2020; but see also Hauser et al., 2007). According to these studies’ findings, it has been suggested that people living in East Asian countries are more reluctant to sacrifice one person in the moral dilemma than their Western European counterparts. A leading hypothesis that could explain these cultural variations is the difference in relational mobility (Awad et al., 2020), especially the difference in the importance of reputation in socio-ecological environments (Yamamoto and Yuki, 2019). Yamamoto and Yuki focused on how actions (i.e., taking action and pulling the lever) and inactions (i.e., doing nothing and not pulling the lever) in the trolley problem influenced individuals’ potential reputation. Action entails the possibility of receiving more positive and negative reputations from others compared to inaction (DeScioli et al., 2011). If we emphasize the socio-ecological explanation here, it is essential to consider that societal or cultural differences exist regarding the category of reputation one must maintain. In low relational mobility societies (see Yuki and Schug, 2020), avoiding accumulating a negative reputation and thereby being disliked and excluded by close relatives are critical for survival and success than in high relational mobility societies (see also Yamagishi et al., 2008; Hashimoto and Yamagishi, 2015). The Japanese demonstrate they do not expect as much positive reputation from taking action (i.e., adopting utilitarian judgments) as Americans do (Yamamoto and Yuki, 2019). Based on the socio-ecological approach, it follows that Japanese people who live in a low relational mobility society, or an interdependent culture, are less likely to adopt utilitarian judgments. The reason for this is not that they are more likely to make deontological judgments, but that they do not adopt utilitarian judgments to avoid the responsibility (or the negative reputation that may result) of taking action. Thus, we speculate that the percentage of adopting utilitarian judgments among Japanese samples is lower than in previous studies. The reason for this may not be the predominance of deontological judgments. Instead, it may be the psychological unwillingness to assume responsibility for acting, thus leading to utilitarian judgments.

In summary, the current study’s purpose is to examine whether the explanation of moral dilemmas based on the dual-process theory is culturally universally applicable. To this end, we focus on the potential influence of decision-making time on people’s moral judgments and hypothesize that the effect of decision-making time can also be applicable even in an interdependent Japanese culture. More specifically, utilitarian judgments will decrease under time pressure, consistent with the dual-process theory of moral judgment (*Hypothesis 1*). We also assumed that the percentage of people who adopt utilitarian judgments is lower among Japanese individuals than in previous studies developed mainly in Western countries. This tendency is not due to the predominance of deontological judgments but results from a psychological unwillingness to assume responsibility for taking action (*Hypothesis 2*). To test these hypotheses, we conducted the study to manipulate decision-making time in the standard trolley dilemma to compare differences under three conditions: intuitive judgment, deliberative judgment, and judgment after a group discussion.

## Materials and Methods

To test Hypothesis 1, we manipulated decision-making time in the trolley dilemma by comparing intuitive and deliberation processes. To examine the deliberation process more carefully, we also utilized a group discussion and exploratory examination of how the discussion can change people’s moral judgments. For example, expressing one’s opinion in a group discussion can lead to greater concern about what others think or feel. Therefore, the current study also examined fickle judgments in moral dilemmas by utilizing group discussion. To test Hypothesis 2, we attempt to administer a new psychological scale measuring people’s utilitarian thinking, deontological thinking, and psychological unwillingness to assume responsibility. Thus, we explore the psychological factors deeply involved in Japanese people’s moral judgments.

### Participants

One hundred and nineteen female Japanese undergraduates (mean age = 19.17 years, *SD* = 0.92) participated in this study. The participants were recruited from a lecture on introductory evolutionary psychology. Participants were informed that the decision to participate was voluntary and that they could stop participating at any point in the study. All students who attended the lecture agreed to participate.

### Procedure

The experimenter first distributed the instruction sheet to all the participants. Then, the standard trolley dilemma was briefly summarized and demonstrated using PowerPoint slides with some illustrations. Furthermore, the experimenter read the summary as shown below for all participants.

*“You are standing on the side of the tracks. A runaway train with broken brakes is rushing in your direction, and you see five people tied to the tracks. If you do nothing, the five people will be run over by the train and shall die. Fortunately, there is a lever on your side. If you pull it, you can surely divert the runaway trolley onto the sidetrack. However, one person is tied to the branch line. If the direction of the train is changed, the person will die. Do you think you should pull the lever? Or do you think you should do nothing and leave the five people to die?”*

After reading the above summary, participants were asked to note their judgment of whether they thought they should pull the lever in this situation within 5 s (the ***intuition*** condition); participants ticked one of six possible answers: “I absolutely think I should not pull it,” “I think I should not pull it,” “If anything, I think I should not pull it,” “If anything, I think I should pull it,” “I think I should pull it,” and “I absolutely think I should pull it.” Next, participants were asked to complete a 10-item questionnaire to examine how strongly they agreed with various thoughts regarding the trolley dilemma issue. This questionnaire was newly developed and administrated by us to distinguish the core principle, that is, utilitarian thinking (e.g., “I think it’s better to save five lives than one.”), deontological thinking (e.g., “I think it’s better to protect a person’s dignity.”), and unwillingness to assume responsibility (e.g., “I don’t want to be responsible for harming one person.”). These items were based on the assumption that the stronger the degree of utilitarian thinking, the more likely the decision will be to pull the lever. Conversely, the stronger the degree of deontological thinking or unwillingness to assume responsibility, the less likely that the lever would be pulled (Table 1 displays the 10-items).

**TABLE 1:** Factor loadings of the subscales of the thinking scale regarding the trolley dilemma issues.

Subscale/Item	Factor 1	Factor 2	Factor 3	Communality
**Factor 1: Deontological thinking (I: α = 0.77, GD: α = 0.88)**				
It is better to protect the dignity of one person.	0.71/0.82	–0.20/–0.16	0.02/0.21	0.40/0.50
One person should not be victimized to save five others.	0.70/0.60	0.07/0.21	–0.12/–0.27	0.61/0.75
It is not good to take away the human right of one person.	0.68/0.95	0.01/–0.01	0.17/0.07	0.43/0.86
Pulling the lever is violating one’s basic human right.	0.64/0.80	0.13/0.12	0.04/–0.09	0.50/0.83
**Factor 2: Unwillingness to assume responsibility (I: α = 0.75, GD: α = 0.87)**				
I do not want to be responsible for victimizing one person.	–0.17/–0.18	1.09/0.98	0.08/–0.02	1.00/0.79
I am likely to regret victimizing one person.	0.15/0.06	0.54/0.86	–0.07/0.12	0.42/0.74
I cannot sacrifice one person because of my personal decision.	0.32/0.08	0.35/0.75	–0.09/0.02	0.38/0.62
**Factor 3: Utilitarian thinking (I: α = 0.75, GD: α = 0.79)**				
It is better to save five lives than one.	0.06/0.18	–0.08/–0.01	0.76/0.78	0.60/0.56
It is better for society that five people survive than one.	0.14/0.11	0.15/0.06	0.75/0.77	0.50/0.54
The sacrifice of one person is unavoidable.	–0.14/–0.15	–0.09/0.05	0.64/0.75	0.53/0.62

*“I” represents the intuition condition, and “GD” represents the group discussion condition. The order of the items is in accordance with the results of the intuition condition.*

After answering these questionnaire items, participants were asked to make the same moral judgment again with no time restrictions (the deliberation condition). This procedure has a lot in common with the so-called “two-response paradigm,” developed to distinguish and compare intuitive judgments from deliberative judgments (e.g., Thompson et al., 2011; Bago and De Neys, 2019). After the participants answered all the questions, they were instructed to put their questionnaires into an envelope. After confirming that all the participants had finished answering the questionnaire, the experimenter asked them to form groups of three or four people. The participants were also asked to exchange their opinions in their groups, such as whether they should pull the lever. We distributed a worksheet to each group to check whether the participants had exchanged views. The participants were asked to explain why they thought the lever should be pulled. The groups included acquaintances, friends of the participants, and individuals who had never met each other. As this experiment emphasized the exchange of opinions with others, we asked the participants to form groups of three or four people regardless of whether they knew each other or had never met before. There were 36 groups in total, and all groups engaged in discussion for approximately 5 min. After the group discussion, participants were asked to make the same decision again (the ***group discussion*** condition). There were six possible answers, as in the intuition and deliberation conditions. After the group discussion and making a third decision, the participants answered the 10-item questionnaire again, concluding the experiment. The entire experiment took approximately 40 min.

### Hypothesis Testing

The purpose of the current study is to examine how people’s judgments in moral dilemmas change when they use their intuition and deliberation and discuss with others. There were six possible answers; therefore, based on participants’ responses, we used a binary variable (i.e., the utilitarian judgments to pull the lever or the deontological judgments not to pull it) for the analysis, as well as assigned each a quantitative variable from 1 (“I absolutely think I should not pull it.”) to 6 (“I absolutely think I should pull it.”) and analyze the change between the conditions from these two indicators. To this end, an analysis of variance and *post hoc* multiple comparison tests were conducted. First, an exploratory factor analysis with Promax rotation was conducted to evaluate the questionnaire’s internal reliability. Then a multiple regression analysis was performed to determine people’s judgments depending on each condition. Finally, the subscale scores of the questionnaire were used as the independent variables and moral judgments in each condition as the dependent variables.

## Results

**FIGURE 1:**
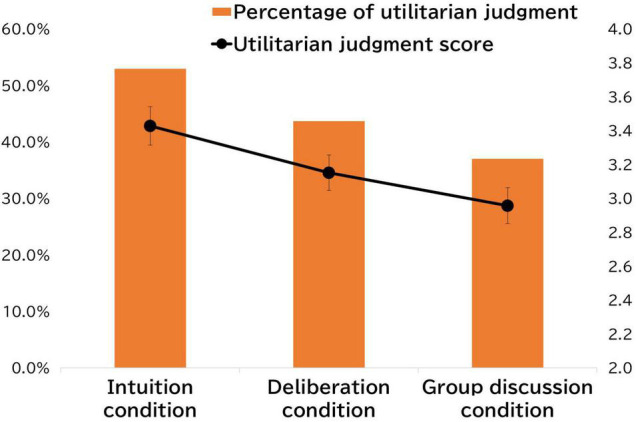
The fickle moral judgment for the conditions using two indicators.

Changes in judgments between the three conditions (intuition, deliberation, and group discussion) are shown in Figure 1. The results demonstrate that utilitarian judgments decreased from 52.9% (*mean* = 3.43, *SD* = 1.24) in the intuition condition to 43.7% (*mean* = 3.15, *SD* = 1.15) in the deliberation condition, and then to 37.0% (*mean* = 2.96, *SD* = 1.15) in the group discussion condition. An analysis of variance, with condition as the independent variable and the mean scores of each utilitarian judgment as the dependent variable, shows the main effect of condition [*F*(2,236) = 17.08, *p* < 0.001, partial η^2^ = 0.13]. The additional multiple comparison analysis shows that there is a significant difference between the intuition and deliberation conditions [*t*(118) = 3.59, *p* < 0.001], the intuition and group discussion conditions [*t*(118) = 4.86, *p* < 0.001], and the deliberation and group discussion conditions [*t*(118) = 2.94, *p* < 0.01]. Given that deliberation is more likely to work better over time, these results contradict previous research (e.g., Suter and Hertwig, 2011) because they suggest that deliberation impedes utilitarian judgments.

**FIGURE 2:**
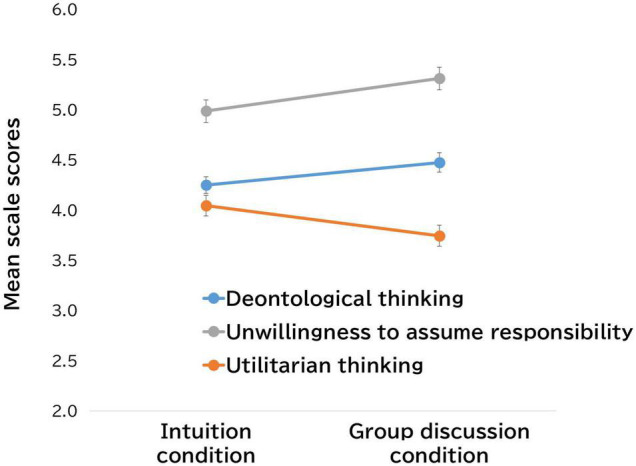
The change in mean scale scores regarding the trolley dilemma.

The thinking scale regarding the trolley dilemma issue that we newly administrated was also analyzed to explore the patterns described above in more detail. As noted in the Procedure section above, this scale was administered after answering the trolley dilemma question in the intuition and the group discussion conditions, respectively. As predicted, the analysis yields three factors. We name Factor 1 “Deontological Thinking,” Factor 2, “Unwillingness to Assume Responsibility,” and Factor 3 “Utilitarian Thinking.” The subscale factor loadings are presented in Table 1. The mean scores of these subscales after the intuition and group discussion conditions are shown in Figure 2. As shown in this figure, the mean scores of the Unwillingness to Assume Responsibility Scale are high and increase over time [*t*(118) = 3.93, *p* < 0.001]. Deontological Thinking Scale scores also show increased scores over time [*t*(118) = 3.38, *p* < 0.001]; conversely, the Utilitarian Thinking Scale scores show a downward trend [*t*(118) = 3.74, *p* < 0.001]. These results are consistent with the pattern shown in Figure 1.

As shown in Table 2, the multiple regression results consistently demonstrate that the higher the score on the Utilitarian Thinking Scale, the more likely one is to adopt utilitarian judgments (intuitive judgments: βs ≥ 0.40, *p* < 0.01; deliberative judgments: βs ≥ 0.44, *p* < 0.01; judgments after group discussion: βs ≥ 0.42, *p* < 0.01), although this result itself was not surprising. Comparatively, the higher the score on the Unwillingness to Assume Responsibility Scale (intuitive judgments: βs ≤ –0.25, *p* < 0.01; deliberative judgments: βs ≤ –0.23, *p* < 0.01; judgments after group discussion: βs ≤ –0.24, *p* < 0.01), the more likely participants are to adopt deontological judgments. However, there are no consistent and significant effects for the Deontological Thinking scale.

**TABLE 2:** Regression analyses to predict intuitive judgments, deliberative judgments, and the judgments after group discussion by the thinking scale regarding the moral dilemma.

		*M* (*SD*)	Intuitive judgments	Deliberative judgments	Judgments after group discussion
After intuition condition	Deontological thinking	4.25 (0.91)	–0.02	–0.10	–0.06
	Unwillingness to assume responsibility	4.99 (1.23)	−0.41**	−0.33**	−0.24**
	Utilitarian thinking	4.04 (1.12)	0.47**	0.44**	0.42**
		*R* ^2^	0.48**	0.41**	0.30**
After group discussion condition	Deontological thinking	4.47 (1.06)	–0.04	–0.12	−0.20*
	Unwillingness to assume responsibility	5.31 (1.21)	−0.25**	−0.23**	−0.24**
	Utilitarian thinking	3.74 (1.14)	0.40**	0.48**	0.55**
		*R* ^2^	0.28**	0.38**	0.55**

**p < 0.01, *p < 0.05.

*Standardized regression coefficients (β’s) are demonstrated.*

## Discussion

Previous studies based on the dual-process model have assumed that the intuitive process increases deontological judgments. In contrast, deliberative reasoning promotes utilitarian decisions (e.g., Greene et al., 2001). Therefore, there seems to be an attempt to save several lives at the expense of a few others in a deliberative manner. This understanding is consistent with some previous findings (e.g., Suter and Hertwig, 2011) demonstrating that moral judgments have been influenced by manipulations of decision-making time; specifically, the deontological judgments were more pronounced under time pressure. If the arguments of previous studies are valid, results would conspicuously show deontological judgments through an intuitive process. Therefore, the current study attempted to examine the validity of this argument and found contradictory patterns. Deliberation makes it more challenging to make utilitarian judgments; thus, Hypothesis 1 is not supported. Additional analysis suggests that a decrease in utilitarian judgments may be related to psychological unwillingness to assume responsibility for the lives of others rather than to an increase in deontological judgments. Thus, Hypothesis 2 is partly supported. These results suggest that Japanese individuals living in an interdependent culture may not pull the lever because of their deontological thinking. Instead, they do not take action because of their unwillingness to assume this responsibility, which suggests that the label of “deontological” judgments may be inappropriate.

The current study’s findings may contradict previous studies but are entirely consistent with the claim that utilitarian judgments can be intuitively generated (Bago and De Neys, 2019). As Białek and De Neys (2017) also point out, some empirical studies showed that intuitive utilitarianism is by no means an exceptional case. Our contention from the current results is that how fickle moral judgments are through people’s deliberation would be related to the nature of society (e.g., Yamagishi and Hashimoto, 2016). Specifically, our findings imply that social environments where people are particularly concerned about the negative publicity of others can modify utilitarianism insofar as utilitarian judgments can lead to negative reactions from others. Although more research findings are needed to examine the implications of the current study, at the very least, our results suggest that when deliberation changes moral judgment, we must also consider the evaluations of those around us that moral judgment brings.

The current results also suggest that the potential responsibility of Japanese individuals’ actions in moral dilemmas may be emphasized (or may include East Asians) compared to Westerners. To illustrate, recent studies (e.g., Awad et al., 2020) suggest that East Asians are more resistant to sacrificing one person in a typical trolley problem. However, the explanation as to why such cultural differences arise has yet to be adequately explained. Inspired by Yamamoto and Yuki (2019), the current study focuses on the potential reputation that actions or inactions in moral dilemmas bring and emphasizes that psychological unwillingness may be the reason why utilitarian judgments are retained in the trolley problem. This explanation seems plausible. Many studies demonstrate that Japanese (or East Asians) tend to adopt strategies that meet the expectations of others as a default instead of behaving according to their preference because they are concerned about negative reputations among others (Yamagishi et al., 2008, 2012; Hashimoto and Yamagishi, 2013, 2016). Although this socio-ecological factor-based explanation sounds plausible, future study is needed to determine whether this explanation is valid.

Although this study yields important insights, several limitations need to be addressed. First, a more effective way to examine the differentiation between intuition and deliberation should be developed. We tested the dual-process model with a within-participant factorial design in the current study. However, by asking the same questions repeatedly, additional confounding factors, excluding intuition and deliberation, may have been included in the participants’ answers. Thus, future research should implement a between-participant factorial design to overcome this limitation. Second, since the current study was conducted at a women’s university, the sample was extremely limited to young Japanese female students. It is possible that men and women differ in their propensity to endorse moral utilitarianism (Fumagalli et al., 2010; Youssef et al., 2012; Lotto et al., 2014; Arutyunova et al., 2016), although it is also suggested that the difference exists only in personal, but not in impersonal moral dilemmas (Friesdorf et al., 2015; Capraro and Sippel, 2017). It must be noted that such a limited sample may have resulted in very few utilitarian responses (slightly above 50%). Therefore, future studies with a broader range of subjects should be done.

Despite these limitations, the current study contributes to understanding culture-specific moral judgments. As the results suggest, East Asians may make moral judgments in a way that avoids responsibility for taking action; thus, interpreting inaction in the trolley problem as deontological judgments must be reviewed. An integrative study of cultural and evolutionary psychology based on an adaptive perspective would be a useful way to test these possibilities.

## Data Availability Statement

The raw data supporting the conclusions of this article will be made available by the authors, without undue reservation.

## Ethics Statement

The studies involving human participants were reviewed and approved by Yasuda Women’s University. The patients/participants provided their written informed consent to participate in this study.

## Author Contributions

HH wrote the whole part of the manuscript. All authors contributed to the study design, collected and analyzed the data, contributed to this article, and approved the submitted version.

## Conflict of Interest

The authors declare that the research was conducted in the absence of any commercial or financial relationships that could be construed as a potential conflict of interest.

## Publisher’s Note

All claims expressed in this article are solely those of the authors and do not necessarily represent those of their affiliated organizations, or those of the publisher, the editors and the reviewers. Any product that may be evaluated in this article, or claim that may be made by its manufacturer, is not guaranteed or endorsed by the publisher.
